# Drug‐Bearing Supramolecular MMP Inhibitor Nanofibers for Inhibition of Metastasis and Growth of Liver Cancer

**DOI:** 10.1002/advs.201700867

**Published:** 2018-06-10

**Authors:** Yujie Ji, Yanyu Xiao, Liu Xu, Jiayu He, Chen Qian, Weidong Li, Li Wu, Rui Chen, Jingjing Wang, Rongfeng Hu, Xudong Zhang, Zhen Gu, Zhipeng Chen

**Affiliations:** ^1^ Department of Pharmacy Nanjing University of Chinese Medicine Nanjing 210023 China; ^2^ Jiangsu Key Laboratory for Functional Substance of Chinese Medicine Nanjing 210023 China; ^3^ State Key Laboratory Cultivation Base for TCM Quality and Efficacy Nanjing University of Chinese Medicine Nanjing 210023 China; ^4^ Department of Pharmacy China Pharmaceutical University Nanjing 210009 China; ^5^ Key Laboratory of Xin'an Medicine Ministry of Education Anhui Province Key Laboratory of R&D of Chinese Medicine Anhui University of Traditional Chinese Medicine Hefei Anhui 230038 China; ^6^ Joint Department of Biomedical Engineering University of North Carolina at Chapel Hill and North Carolina State University Raleigh NC 27695 USA

**Keywords:** doxorubicin, liver cancer, metastasis, matrix metalloproteinase (MMP) inhibitor, peptide drug conjugates

## Abstract

Treatment of hepatocellular carcinoma (HCC) requires sustained suppression of tumor cell growth and metastasis for long‐term efficacy. However, traditional intratumoral drug delivery system always exhibits burst release with less therapeutic outcomes. Here, a new self‐assembling amphiphilic peptide drug conjugate (SAAPDC) is fabricated as a “two‐in‐one” nanofiber system comprising a hexapeptide as a matrix metalloproteinases (MMP) inhibitor and doxorubicin (DOX) for the treatment of HCC. The results indicate that doxorubicin‐conjugated peptide (DOX‐KGFRWR) self‐assembles to form long nanofibers showing sustained release property for inhibiting the enzymatic activities of MMP‐2 and MMP‐9. This nanofiber not only inhibits tumor growth in situ but also effectively prevents pulmonary metastasis in an SMMC7721 cell line–based mouse model. In summary, this hexapeptide‐based supermolecule system represents a promising nanoscale platform to sustain drug release with high loading capacity for intratumoral administration. Moreover, the delivery of chemotherapeutic drugs via drug‐bearing supramolecular MMP inhibitor nanofibers simultaneously inhibits metastasis and tumor growth to achieve synergistic effects for metastatic HCC therapy.

## 1 Introduction

The peptide has great potential for their use in biological and biomedical applications due to its intrinsic low toxicity.^1^ More importantly, peptide can self‐assemble into various nanostructures, such as nanoparticles, nanofiber, nanogel.^2^ Among the various peptide nanostructures, peptide nanofiber with multifunction properties are highly sought because of their promising biodegradability. However, peptides nanofibers have limited functionality that restricts their applicability as therapeutic effect. Recently, peptide drug conjugates attracted a widespread attention as localized drug delivery systems.^3^ Localized drug delivery systems offer several advantages over systemic administration, including the ability to achieve high drug concentrations, increased drug retention time, reduced systemic side effects, and a decreased total drug cost.^4^ Intra‐articular therapy is now an accepted treatment modality in patients with clinical arthritis, particularly when treatment with nonsteroidal agents has failed. In addition, several studies have reported the clinical application of chemoembolization to improve the survival rate of liver cancer patients. Open surgery for implantation is always needed to treat malignant glioma.^5^ However, traditional local administration (implant, wafer, etc.) is strictly limited by the reliance on surgical implants, resulting in a poor ability to control drug release and single functionality. In contrast to surgical implant systems, injectable gels present great benefits because they allow for minimally invasive drug delivery directly to the diseased tissue.

Recently, some studies have focused on developing new drug delivery systems to improve the efficacy of localized drug delivery systems. Among these systems, self‐assembling amphiphilic peptide drug conjugates (SAAPDCs) have received great attention for medical applications due to their inherent biocompatibility and biodegradability.^6^ SAAPDCs increase local drug concentrations, improve treatment efficiency and lengthen the period of drug efficacy, reduce systemic toxicity, improve patient compliance, and enable synergistic therapy. Over the past decade, Cui et al. have designed and synthesized a broad range of amphiphilic molecules to create self‐assembling biomaterials for cancer and arthritis therapeutics.^7^ In addition, the Xu and co‐workers have reported the design and synthesis of hydrogelators composed of d‐amino acids and a nonsteroidal anti‐inflammatory drug for the development of multifunctional supramolecular hydrogelators that exhibit excellent selectivity for inhibiting cyclooxygenase‐2 (COX‐2).^8^

However, much of the current effort in oncotherapy has focused on combination therapy due to the molecular complexity of cancer. Hepatocellular carcinoma (HCC) is the third main cause of cancer‐related mortality in men worldwide. Metastasis is one of the main causes of HCC recurrence and is the primary cause of cancer‐related death in the clinic.^9^ The most common sites for HCC metastasis are the lung, bone, and brain.^10^ Most patients who eventually die from HCC display pulmonary metastases. Hence, the inhibition of pulmonary migration may represent a preferred therapeutic strategy for metastatic HCC. HCC metastasis was reported to require the abrogation of cell–cell contacts and the remodeling of the extracellular matrix and cell–matrix interactions,^9^, ^11^ as evidenced by reduced E‐cadherin expression and the increased expression of matrix metalloproteinases (MMPs) and other proteins. MMPs are zinc‐dependent endopeptidases that have a similar structure. Collectively, these enzymes can degrade every component in the extracellular matrix (ECM). Unequivocal data demonstrates that levels of MMP‐2 and MMP‐9 increase with the progression of liver tumors.^12^ Preliminary attempts to develop MMPs as a chemotherapy target by utilization of synthetic MMP inhibitors (MMPIs) to prevent cancer invasion. MMPIs reduced tumor growth and invasion in vitro and in vivo. However, MMPIs achieved disappointing results in Phase I, II, and III clinical trials for a variety of reasons.^13^ Despite these disappointing clinical results with MMPIs, MMPs may still represent an excellent target for chemotherapeutic delivery if an alternate prodrug‐like approach is used. Hence, MMPIs has a potential role in blocking cancer progression, but simultaneously may suppress normal tissue function or host defense processes.

Hence, we hypothesized that a combination drug delivery system based on in situ entrapment in a SAAPDC nanofiber would achieve high drug loading, improve the thermodynamic stability of SAAPDC, prolong drug release, enhance the anticancer activity of the drug, and inhibit tumor migration. Many peptides have strong metal coordination abilities, particularly basic amino acids. For example, Zhang and co‐workers reported the preparation of dipeptide nanoparticles by self‐assembly with zinc ions and their use in imaging and monitoring drug release in real time.^14^ Hence, we hypothesized that peptides are useful to deliver MMPIs due to their chelating ability.

**Scheme 1:**
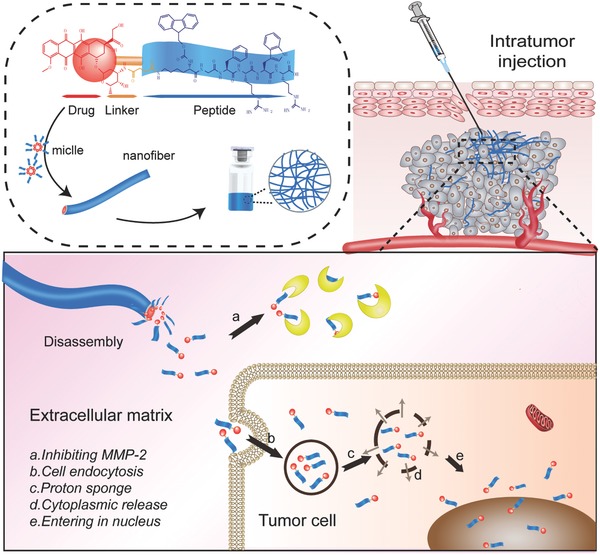
Schematic fabrication of multifunctional SAAPDC “two‐in‐one” nanofiber systems with inhibiting metastasis and growth of liver cancer, possessing an ultrahigh drug‐loading, long‐acting sustained‐release and simultaneously inhibiting MMP‐2.

Here, we designed and prepared SAAPDC “two‐in‐one” nanofiber systems that inhibit the growth and metastasis of liver cancer, possess ultrahigh drug loading, and allow for long‐lasting sustained‐release (**Scheme** **1**). First, an amphiphilic peptide drug conjugate was synthesized from oligomeric peptides (KGFRWR) derived from amyloid β‐protein^15^ and doxorubicin (DOX) for the development of a supramolecular nanofiber with excellent selectivity for inhibiting MMP‐2 and MMP‐9. This initial liquid formulation of the amphiphilic peptide drug conjugate flowed easily at room temperature, but formed a nanofiber in the diseased region after administration. The prodrug was slowly released from the nanofiber via both the diffusion and degradation of the amphiphilic peptide drug conjugate, leading to the inhibition of MMP activity and proliferation of liver cancer. This delivery system altered the distribution of the drug, increased the local concentration in the liver cancer region, decreased the drug distribution in other tissues, reduced the adverse cardiotoxicity, and prolonged the retention of the drug in the target area.

## 2 Results and Discussion

### 2.1 Preparation and Characterization of DOX–KGFRWR

The conjugation of suitable amino acids to antitumor drugs such as DOX was shown to significantly improve cellular uptake and drug resistance compared to the free drug.^16^ We conjugated DOX to the backbone of the hexapeptide KGFRWR to generate nanofibers in situ (or in vivo) and evaluate the correlation between the structure and activity of DOX nanofibers. Figure S1 in the Supporting Information shows the molecular structures of the KGFRWR and DOX–(Fmoc) KGFRWR (DOX–KGFRWR) designed in this work. The hexapeptide KGFRWR possesses alternating hydrophobic–hydrophilic side‐chain functionalities that form nanofiber structures when conjugated to a larger hydrophobic moiety, and even form nanofibers. Conjugation of the hydrophobic DOX to the N‐terminus of this peptide is expected to result in a similar self‐assembly behavior.

**Figure 1:**
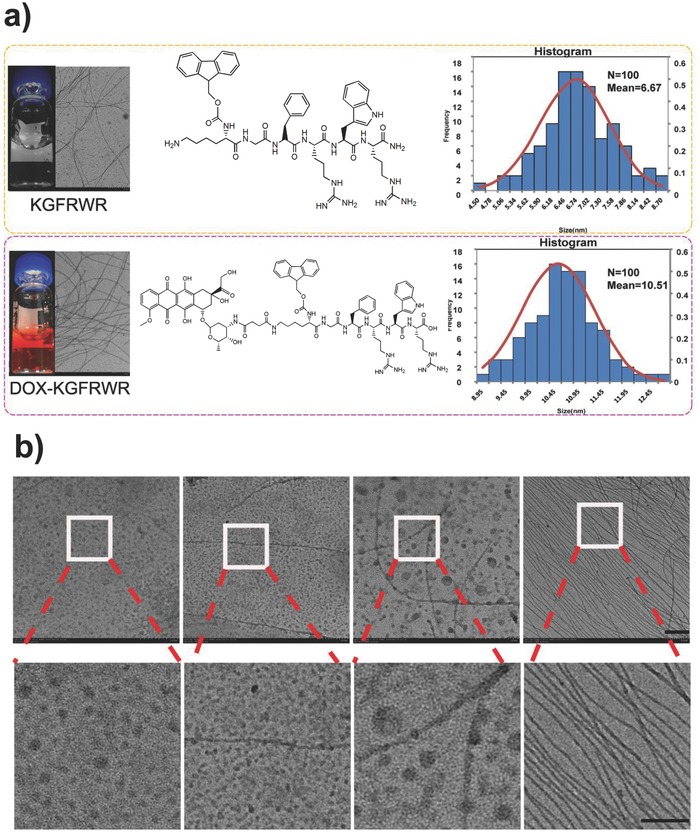
a) The normal distribution of DOX–KGFRWR fiber diameters and the principle diagram of the DOX–KGFRWR assembly. b) The TEM images of fiber forming process of DOX–KGFRWR (Scale bar = 200 nm).

DOX–KGFRWR was synthesized using standard solid‐phase Fmoc peptide synthesis protocols,^17^ as shown in Figure S1 in the Supporting Information, and was characterized by high performance liquid chromatography, mass spectrometry (MS), and UV–vis spectroscopy (Figures S2 and S3, Supporting Information). First, the critical aggregation concentration (CAC) was determined using pyrene as a fluorescent probe.^18^ According to the curve of the fluorescent intensity ratio (*I*_1_/*I*_3_) of pyrene versus DOX–KGFRWR concentration in water, the CAC value for DOX–KGFRWR was ≈50 × 10^−6^ m, indicating that DOX–KGFRWR molecules start to aggregate into nanoparticles at concentrations greater than 50 × 10^−6^ m (Figure S4, Supporting Information). Then, we investigated the assembly process of DOX–KGFRWR in detail. As shown in Figure S5 in the Supporting Information, DOX–KGFRWR first self‐assembled into nanoparticles in 6 h, then partially transitioned into a mixture of nanoparticles and nanofibers from 12 to 24 h, and finally completely transformed into nanofibers after 48 h. However, KGFRWR gradually formed into nanofibers without micellar process during 6 h, and then also completely transformed into nanofibers after 48 h. According to the results of the gelation test, KGFRWR and amphiphilic DOX–KGFRWR conjugates formed stable, colorless transparent, or red nanofibers, respectively, at a concentration of 1.5 × 10^−3^ m (**Figure** **1**). Transmission electron microscopy (TEM) was used to examine the DOX–KGFRWR nanofibers and evaluate the characteristics of the respective molecular assemblies. KGFRWR and DOX–KGFRWR self‐assembled to form long nanofibers with average widths of 6.67 and 10.51 nm (Figure a), respectively, where the difference in the diameter between the two nanofibers is approximately the size of DOX. The nanofibers may form due to the inherent chirality of the hexapeptide. The hydrophobic tryptophan group in the predominantly hydrophilic plane may facilitate the packing and lead to an increase in the interfacial curvature, favoring a fiber‐like structure at concentrations exceeding the critical aggregation concentration.

### 2.2 In Vitro Cytotoxicity Induced by DOX–KGFRWR

**Figure 2:**
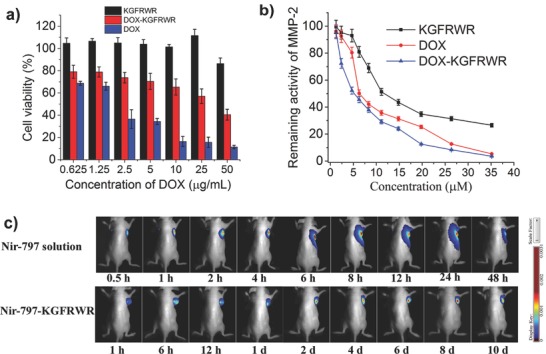
Release characteristics and biodistribution in vivo of the DOX–KGFRWR nanofibers. a) In vitro inhibition rate of DOX–KGFRWR, DOX, and KGFRWR at different concentrations against SMMC7721 cells after 24 h incubation. b) The Inhibition of MMP‐2 by KGFRWR, DOX, and DOX–KGFRWR. c) In vivo real‐time fluorescence images of SMMC‐7721 xenograft‐bearing mice were taken at different time after intratumor injection of Nir‐797 solution and Nir‐797‐KGFRWR assembly. Data are given as the mean ± SD (n = 6).

The 3‐(4,5‐dimethylthiazol‐2‐yl)‐2,5‐diphenyltetrazolium bromide (MTT) assay was used for quantitative testing of the viability of SMMC7721 cells treated with KGFRWR, DOX, and DOX–KGFRWR conjugates. As shown in **Figure** **2**a, the blank KGFRWR peptide had no obvious effect on cell viability at concentrations ranging from 0.625 to 50 µg mL^−1^. Over the same concentration range, the DOX–KGFRWR conjugate dramatically inhibited SMMC7721 cell growth, although the inhibition ratio was inferior to free DOX. The IC_50_ values for DOX–KGFRWR and DOX were 34.55 and 1.888 µg mL^−1^, respectively. Thus, DOX–KGFRWR was readily internalized through the diffusion and endocytosis of the cationic peptide.

### 2.3 MMP‐2 Inhibitor Screening Assay

To support our hypothesis, KGFRWR conjugates must be able to effectively inhibit the action of the MMP‐2 enzyme, an enzyme involved in tumor cell migration. As illustrated in Figure b, we therefore performed in vitro MMP‐2 enzymatic inhibition assays to evaluate the inhibitory efficacy of the KGFRWR peptide with coordination group. The IC_50_ values for KGFRWR, DOX, and DOX–KGFRWR against the MMP‐2 enzyme were 14.19, 8.68, and 5.27 × 10^−6^ m, respectively. As the reported IC_50_ value for an MMPI (e.g., batimastat) against MMP‐2 is 4 × 10^−9^ m,^20^ the IC_50_ values for KGFRWR and DOX–KGFRWR indicate a reasonable level of MMP‐2 inhibition, given the intended local delivery application. Fortunately, the attachment of the small hexapeptide to DOX strengthens its inhibition of MMP‐2 activity, which may be due to the exposure of the DOX coordination group. These results validate the use of the DOX–KGFRWR conjugate as a candidate for injectable DOX nanofiber, indicating that the KGFRWR hexapeptides not only lead to self‐assembly and gelation but also provide beneficial inhibition of enzymatic activity.

### 2.4 The In Vitro Release Profile and Pharmacokinetics of DOX–KGFRWR Nanofibers

We evaluated the sustained release of DOX from the DOX–KGFRWR nanofiber to evaluate the long‐lasting effects of this system. We incubated DOX solution or DOX–KGFRWR nanofiber with 10 mL of phosphate buffer saline (PBS) buffer solution (pH 7.4) in a dialysis bag at 37 °C for 12 d; the buffer was refreshed and monitored at predetermined times. Figure S6a in the Supporting Information shows the release profiles of DOX and DOX–KGFRWR nanofibers. The release rate of the DOX solution was faster than the DOX–KGFRWR nanofiber. Dynamic light scattering measurements of the solution released from the nanofiber exhibited little difference from the DOX solution (data not shown). Six male rats were administered 200 µL of DOX solution or DOX–KGFRWR nanofibers via an intratumor injection to evaluate the DOX–KGFRWR nanofibers in an in vivo environment. The dynamic behavior of the nanofiber was monitored at predetermined intervals after injection by liquid chromatography‐tandem mass spectrometry (LC/MS/MS). The DOX–KGFRWR nanofiber clearly achieved sustained release of DOX over a long period of time, maintaining a nearly steady concentration in the plasma with no burst release characteristics (Figure S6b and Table S1, Supporting Information). In addition, tumor in the mice injected with Nir‐797–KGFRWR nanofibers exhibited persistent fluorescent signal compared with Nir‐797 solution. (Figure 2c). Based on these results, these DOX–KGFRWR nanofibers can provide sustained localized drug delivery following intratumoral injection. All of the mouse studies were performed in the context of an animal protocol approved by the committee at Nanjing University of Chinese Medicine.

### 2.5 Endosomal Escape of DOX–KGFRWR

The efficiency of the intracellular delivery of DOX–KGFRWR and DOX in SMMC7721 cells was investigated using confocal laser scanning microscopy (CLSM). Lysosomes displayed green fluorescence, and DOX–KGFRWR and DOX displayed red fluorescence. Colocalization of DOX–KGFRWR and DOX with the specific organelle dye appeared yellow. The overwhelming majority of cells predominantly exhibited yellow fluorescence after a 1 h incubation with DOX–KGFRWR and DOX, indicating that both forms of DOX were readily taken up by the cells through endocytosis. However, DOX–KGFRWR accumulated in the lysosomes rather than being evenly dispersed throughout the cells due to its temporary no escape from lysosomes. A few cells were saturated with red fluorescence when the hepatoma cells were incubated with DOX. Red fluorescence also accumulated in the cell nucleus because DOX tends to traffic to the nucleus. Most importantly, yellow fluorescence accumulated in the lysosomes when the cells were incubated with DOX–KGFRWR, indicating that the conjugate has a limited ability to escape from lysosomes (Figure S7, Supporting Information).

### 2.6 In Vitro Antimetastatic Effects

**Figure 3:**
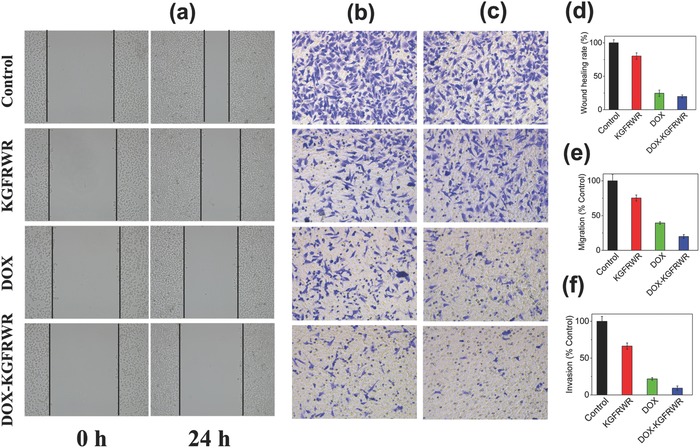
a) The wound healing images and d) quantitative analysis after scratch for 24 h. Microscopy images of b) migration and c) invasion and e,f) quantitative analysis of SMMC7721 cells that passed through the membrane. Cells were preincubated with KGFRWR, DOX, DOX–KGFRWR. Cells without treatments were used as control.

The abilities of DOX–KGFRWR and DOX to inhibit the metastasis of SMMC7721 cells in vitro were investigated. First, a wound healing assay was used to evaluate the inhibitory effects of KGFRWR, DOX, and DOX–KGFRWR on SMMC7721 cell motility. As shown in **Figure** **3**a, the control group of SMMC7721 cells exhibited strong, aggressive motility. KGFRWR slightly reduced wound healing, whereas DOX and DOX–KGFRWR reduced healing to 24.5% and 19.7% of that seen in the control group (Figure 3d), respectively. Thus, DOX–KGWRFR exerted a slightly stronger inhibiting effect on wound healing than DOX. A migration assay was used to further evaluate the inhibitory effects of KGFRWR, DOX, and DOX–KGFRWR on SMMC7721 cell migration. As presented in Figure 3b and e, compared to the control group, 75.2% of cells in the KGFRWR group had migrated into the lower chamber suggesting that KGFRWR exerted a slight inhibiting effect on the migration of SMMC7721 cells, consistent with the wound healing results. Cells treated with DOX and DOX–KGFRWR exhibited markedly decreased migration, with migration rates of 39.4% and 19.9%, respectively, compared with those in the control, indicating that DOX–KGWRFR exerted a stronger inhibiting effect on migration than DOX. Moreover, the results of the invasion assay were consistent with the results of the wound healing and migration assays (Figure 3c and f).

### 2.7 In Vivo Antitumor Effect

**Figure 4:**
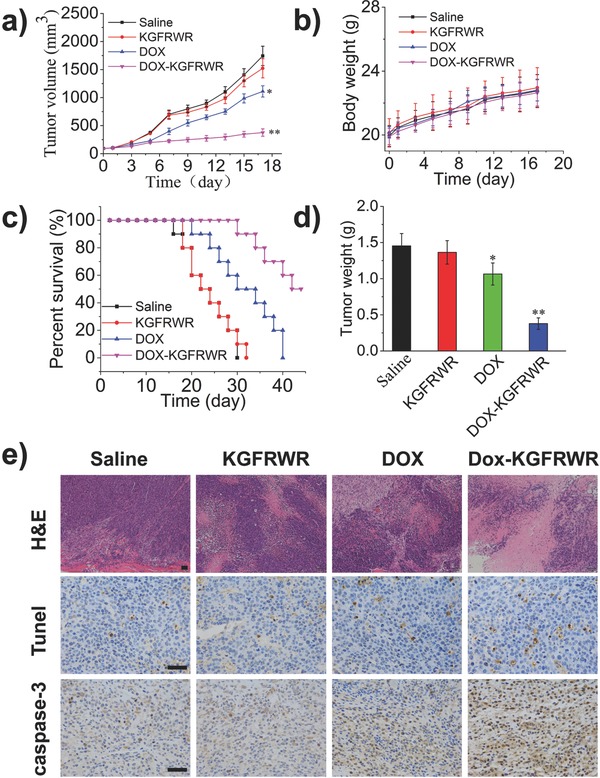
In vivo antitumor effect in SMMC7721 tumor‐bearing mice (n=15). a) The variation profiles of tumor volumes. b) Body weights changes of tumor‐bearing mice (n=15). c) Survival curves of mice bearing SMMC7721 tumors after various treatments (n=10). d) Tumor weight on day 17 after the first administration (n=5). Representative microphotos (×200) of tumor sections stained for e)Tunnel, f) H&E, and g) immunohistochemistry (IHC), respectively (Scale bar = 50 µm).

To monitor the antitumor efficacy and toxicity of DOX–KGFRWR nanofibers, the average tumor sizes and body weights were recorded during the experiment. At the end of the experiment, tumors were photographed and weighed. As shown in **Figure** **4**a, the tumors in the saline group grew so rapidly that the mean tumor volume expanded from 100 to 1800 mm^3^ within 17 d. DOX inhibited tumor growth compared with the saline and KGFRWR groups. The antitumor efficacy of the DOX–KGFRWR nanofiber was superior to all other treatments, with a final tumor volume of 376 mm^3^. The excellent therapeutic efficacy of the DOX–KGFRWR nanofiber was attributed to the controlled distribution and long‐term retention of DOX and the hexapeptide‐enhanced DOX permeation into SMMC7721 tumor cells. None of the treatments led to serious body weight losses (Figure 4b), indicating that the DOX–KGFRWR nanofiber did not induce severe systemic toxicity compared with DOX. And the biodistribution results indicated that the DOX–KGFRWR nanofiber did not induce severe systemic toxicity compared with DOX (Figure S8, Supporting Information). DOX–KGFRWR has significantly prolonged survival rates (Figure 4c). As shown in Figure 4d, the tumor weights and sizes were consistent with the tumor volumes.

Tumors were sectioned, subjected to hematoxylin and eosin (H&E), terminal deoxynucleotidyl transferase(TdT)‐mediated fluorescein‐dUTP (dUTP) Nick‐End Labeling, and immunohistochemical staining, and then observed under a confocal microscope to obtain a better understanding of the antitumor effects at the cellular level. As necrosis naturally occurs because of the internal hypoxia inside the tumor, both H&E and TUNEL images were captured near the periphery of the tumor. As shown in Figure 4e, the degree of apoptosis or necrosis observed in tumor sections was consistent with the tumor volume. Mice treated with saline displayed no significant necrosis or apoptosis in H&E staining (Figure 4e), and the nuclei in all of these cells were complete and dyed dark blue. In mice treated with the DOX–KGFRWR nanofiber, the tumors were predominantly necrotic or apoptotic, consistent with the observed antitumor effect. TUNEL staining (Figure 4e) was performed to characterize apoptosis, and the results were consistent with the H&E staining, indicating that the excellent antitumor efficacy of the DOX–KGFRWR nanofiber induced greater levels of necrosis and apoptosis in cells (Figure 4e/caspase‐3).

### 2.8 In Vivo Antimetastatic Effects

**Figure 5:**
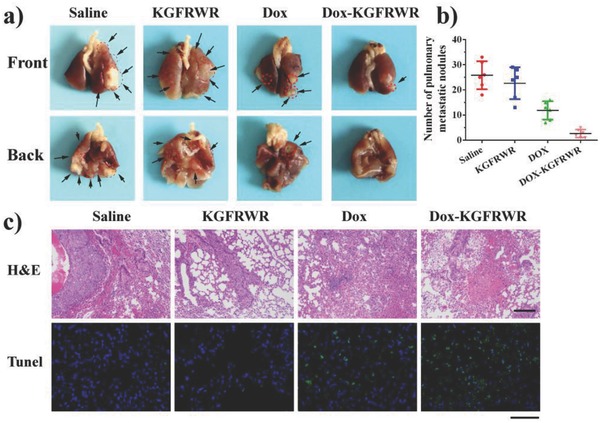
In vivo antimetastasis effects on the SMMC7721 pulmonary metastatic mouse model. a) Representative images of pulmonary metastatic nodules, b) Quantitative analysis of pulmonary metastatic nodules, significance determined by student's t test: **p < 0.01. c) histopathologic examination of the lungs, and tunnel analysis of the lungs from mice bearing hepatic tumors of SMMC7721 cells on day 21 after injection (Scale bar = 200 µm). Data are given as the mean ± SD (*n* = 6).

The antimetastatic effects were evaluated by determining the number of metastatic nodules and the degree of H&E staining in the lung tissues. The saline group exhibited large numbers of tumor metastases on the lung surface (**Figure** **5**a,b), suggesting that SMMC7721 cells exhibited a strong metastatic capacity in vivo. The number of metastatic nodules in the free DOX group was distinctly decreased compared with that in the control, potentially resulting from the antiproliferative effect of DOX. Excitingly, the hexapeptide alone had a slight inhibitory effect on metastasis compared with the control group, indicating that the hexapeptide itself inhibited the metastasis of SMMC7721 cells in vivo without exerting an effect on tumor growth. The DOX‐KGFRWR nanofiber led to synergistic antimetastatic and antitumor growth effects. The minimum number of metastatic nodules observed was tenfold less than the group treated with saline, which was attributed to the better antimetastatic activity of the higher concentration of the hexapeptide itself and the stronger antitumor growth in situ due to the higher concentration of the hexapeptide combined with the same dose of DOX. In addition, the H&E staining and TUNEL images further supported these results (Figure 5c).

## 3 Conclusion

In summary, a new SAAPDC “two‐in‐one” nanofiber system consisting of a MMP inhibitor hexapeptide and an anticancer drug was designed as an intratumor treatment for proliferation and migration. This delivery system possesses ultrahigh drug loading and allows for long‐lasting sustained release. The hexapeptide KGFRWR was conjugated to DOX, resulting in the formation of supramolecular nanofibers with excellent selectivity for the inhibition of MMP‐2 and MMP‐9. This liquid formulation of the amphiphilic peptide drug conjugate flowed easily at room temperature and quickly formed a nanofiber after administration. The prodrug was released slowly from the nanofiber via both diffusion and degradation of the amphiphilic peptide drug conjugate, leading to MMP inhibition and antiproliferative effects on liver cancer. Moreover, the nanofiber altered the distribution of the drug, increased the local concentration in the liver tumor, decreased the distribution to other tissues, reduced its adverse cardiotoxicity, and prolonged the retention of the drug in the target area. Furthermore, based on the results of the in vivo release study, this platform is capable of achieving a steady and sustained drug release profile. This local administration approach may represent a useful strategy for reducing the adverse drug reactions of other therapeutic agents. Moreover, the supramolecular nanofibers also hold potential as a theranostic nanoplatform by grafting other functional groups to enable combined tumor therapy and imaging.

## 4 Experimental Section

*Solid‐Phase Peptide Synthesis (SPPS) of Fmoc‐KGFRWR*: All conjugates were prepared by SPPS using a 4‐methyl‐benzhydrylamine resin (100–200 mesh and 0.88 mmol g^−1^) and Fmoc‐protected amino acids with appropriate side chain protection,^22^ i.e., tert‐butyl groups for acids such as Fmoc‐Lys(Mtt)‐OH. After bubbling with N_2_ in dry dichloromethane (DCM) for 20 min, the resin swelled and was washed with dry *N*,*N*‐dimethylformamide (DMF) (3 × 3 mL). Then, the amino acid was loaded onto the resin through its C‐terminal carboxylate group in a DMF solution of ethyldiisopropylamine (DIEA) and Fmoc‐protected amino acid (2 equiv.) for 0.5 h. The resin was bubbled with the stop solution DCM/methanol (MeOH)/DIEA=16:3:1 to inactivate the unreacted sites for 0.5 h after washing with DMF (3 × 3 mL). Then, the resins were dealed with 20% piperidine DMF solution of piperidine for 30 mins for removal of protective groups, followed by coupling the Fmoc‐protected amino acid (2 equiv.) with free amino group on the resin via DIEA/hydroxybenzotriazole (2 equiv.). The above two steps were duplicated to extend the peptide chain until the final sequence was obtained. The resin was washed with DMF 3–5 times after each step.

*Preparation of COOH‐(Fmoc)KGFRWR*: Fmoc‐K(NH_2_)GFRWR‐Rink (1 mmol) was dissolved in dry DCM, and trifluoroacetic acid (3 mmol) and triisopropylsilane (5 mmol) were added and shaken for 0.5 min. This step was repeated two times. Then, the deprotected K(NH_2_)GFRWR‐Rink peptide was dispersed in DMF, and succinic anhydride (1.5 eq.) and DIEA (2.0 eq.) were added to the above solution and stirred overnight. The obtained COOH‐K(NH_2_)GFRWR‐Rink peptide was dispersed in H_2_O, and trifluoroacetic acid (2.5 mmol) and triisopropylsilane (2.5 mmol) were added and shaken for 3 h. The product was then concentrated in a rotary evaporator under reduced pressure, followed by precipitation (three times) in cold diethyl ether. The product was obtained by filtration, washed with ice‐cold diethyl ether, and dried in a vacuum desiccator.

*Preparation of DOX–KGFRWR*: The COOH‐(Fmoc)KGFRWR peptide (1 mmol) was dissolved in 10 mL of anhydrous DMF, and DOX∙HCl (1 mmol), (3‐hydroxy‐3H‐1,2,3‐triazolo[4,5‐b]pyridinato‐O)tri‐1‐pyrrolidinylphosphonium hexafluorophosphate (1.0 mmol) and DIEA (3.0 mmol) were added and stirred for 2 d. The resulting crude products were deprotected and purified through reverse phase HPLC; the purity was verified by LC/MS. The molecular weight of DOX‐KGFRWR was investigated by matrix‐assisted laser desorption/ionization‐time‐of‐flight mass spectrometry (AB SCIEX TOF/TOF 5800, USA). The UV absorption spectrum of DOX–KGFRWR was detected with a UV–vis spectrophotometer.

*HPLC Analysis of DOX–KGFRWR*: The purity of the synthesized DOX–KGFRWR conjugate was confirmed by HPLC. A Varian PLRP‐S column (250 mm × 4.6 mm, 10 µm) was used to separate analytes. The column temperature was 25 °C and the wavelength was set to 480 nm. The mobile phase consisted of acetonitrile and 0.1% trifluoroacetic acid, and a gradient method was employed for the analysis (30–40% acetonitrile over 0–10 min, 40–50% acetonitrile over 10–20 min, 50–60% acetonitrile over 20–30 min).

*The CAC of DOX–KGFRWR*: The CAC of DOX–KGFRWR was measured by fluorescence spectroscopy with pyene as a fluorescent probe. Fifty microliters of a pyrene solution (1 × 10^−4^ m in methanol) were added to 20 mL vials, and the solvent was removed by natural evaporation. Subsequently, 5 mL aqueous solutions of DOX–KGFRWR with concentrations ranging from 0.05 to 5000 × 10^−6^ m were added to the vials. After a 5 min ultrasonic treatment and 48 h incubation, the emission spectra of the solutions were measured using a spectrofluorophotometer (RF‐5301, SHIMADZU, Japan).

*TEM*: TEM images of the process of DOX–KGFRWR fiber formation were visualized using the following representative procedure. The sample was dropped onto a copper grid (300 mesh) coated with a carbon membrane and then stained with 2% uranyl acetate for 2 min. The grid was allowed to dry before characterization. After air drying, the sample was observed using a TEM/H‐7650 (Hitachi, Japan) operating at 80 kV. Images were captured with a CCD/MT280B.

*Release Profile of DOX–KGFRWR Nanofibers*: DOX–KGFRWR conjugates (3.4 mg) were dissolved in 2 mL of PBS buffer (10 × 10^−3^ m, pH 7.4), allowed to self‐assemble into nanofibers for 3 d, transferred into a preswelled dialysis bag (molecular weight cut‐off = 2000 Da), and immersed in 10 mL of PBS buffer (10 × 10^−3^ m, pH 7.4). The drug release experiment was performed at 37 °C with stirring at 100 rpm. At predetermined times, 0.5 mL of the sample solution was withdrawn and the DOX–KGFRWR concentration was measured using HPLC. The volume of the removed sample was immediately replaced with 0.5 mL of PBS buffer (10 × 10^−3^ m, pH 7.4). The release profile of DOX was used as the control group.

*MMP‐2 Inhibitor Screening Assay*: The MMP‐2 inhibitory activity of the samples was analyzed using a fluorescent substrate method, as previously described.^23^ The MMP substrate peptide MOCAC‐Pro‐Leu‐Gly‐Leu‐Dap(Dnp)‐Ala‐Arg‐NH_2_ was purchased from Sigma. After preincubation of 50 µL of a reaction mixture containing an appropriate amount of MMP‐2 (5 × 10^−9^ m) with 20 µL of test sample solution (KGFRWR, DOX, and DOX–KGFRWR) in microplate wells at 37 °C for 15 min, 10 µL of MMP substrate peptide (50 × 10^−6^ m) were added to initiate the proteolytic reaction. The fluorescence intensity was measured at 328 nm for excitation and 393 nm for emission every 15 min for a 2 h period without interrupting the reaction using a fluorescence microplate reader (POLARstar Omega, Germany). The IC_50_ values were calculated based on the data obtained at 2 h.

*Cell Toxicity Assay*: Cytotoxicity in SMMC7221 cells was quantified using an MTT assay. Cells were incubated in Dulbecco's modified Eagle medium (DMEM) medium containing 10% fetal calf serum at 37 °C in an atmosphere of 5% CO_2_ in air. For the cytotoxicity assay, SMMC7721 cells were precultured in 96‐well cell culture plates at a density of 1.0 × 10^5^ cells per well in 100 µL of medium and grown overnight. Cells were then incubated with various concentrations of DOX–KGFRWR and dox for 24 h. After the appropriate incubation times, 20 µL of a 5 mg mL^−1^ (MTT solution in PBS (pH 7.4) were added to each well. After the cells were incubated for another 4 h, the media were withdrawn and DMSO (150 µL) were added to each well to dissolve the formazan crystals. The each well was then measured at 490 nm by a microplate reader. Cell viability was calculated as the determinatd percentage of viable cells after drug treatment compared with the untreated cells. The IC_50_ values for DOX–KGFRWR were determined from the activity curves, which were measured at five different concentrations of DOX–KGFRWR.

*Endosomal Escape of DOX–KGFRWR*: SMMC7721 cells were seeded in thin glass‐bottomed 35 mm petri dishes at a density of 1.0 × 10^4^ cells per dish and incubated for 24 h at 37 °C in a 5% CO_2_ atmosphere to support reattachment. Next, the media were removed and the cells were washed twice with PBS, followed by an incubation with LysoTracker Red DND‐99 (100 × 10^−9^ m for 1 h) at 37 °C. Then, the freshly prepared DOX–KGFRWR and DOX solutions were added immediately to the petri dishes, followed by an incubation at 37 °C for 1 or 3 h. Afterward, cells were stained with Hoechst 33342 (100 × 10^−6^ m in PBS) for 20 min, fixed with 4% paraformaldehyde, and visualized under a CLSM (FV1000, Olympus).

*Pharmacokinetics of DOX–KGFRWR Nanofibers in Rats*: Twelve male Sprague–Dawley rats (180–220 g) were randomly divided into two groups, and then the rats were administered 200 µL of a DOX solution (containing 1000 µg of DOX) or DOX–KGFRWR nanofiber (containing 1000 µg of DOX) via an intramuscular injection. The dynamic behavior of DOX was monitored at predetermined intervals postinjection (0.17, 0.5, 1, 2, 4, 6, 8, 10, 12, and 24 h, and 1.5, 2, 3, 4, 5, 6, 7, 8, 9 and 10 d) by LC/MS/MS. The rats were anaesthetized with ether. A heparinized capillary was then inserted into eyeground veins to obtain 0.3 mL of blood in a plastic centrifuge tube. Plasma samples were collected by centrifugation at 12 000 rpm for 5 min and then stored immediately at −20 °C until analysis. Next, 300 µL of acetonitrile containing an internal standard (clarithromycin, 20 ng mL^−1^) were added to the 100 µL plasma samples to precipitate protein, the mixture was centrifuged at 13 000 rpm for 5 min, and 50 µL of supernatant were assayed by LC/MS/MS.

*In Vivo Biodistribution of DOX and DOX–KGFRWR*: Sixty male Institute of Cancer Research (ICR) mice were randomly divided into two groups, and then the mice were administered 200 µL of a DOX solution (containing 140 µg of DOX) or DOX–KGFRWR nanofiber (containing 140 µg of DOX) via an intramuscular injection. The tissues of heart, liver, spleen, lung, kidney, and brain were harvested at 0.167, 0.5, 1, 2, 4, and 8 h, respectively. Then washed the tissues with saline and weighed, finally, tissue homogenate was obtained by adding a certain amount of saline. Separate the homogenate from each group by 90 µL in 2 mL centrifuge tube, add 10 µL of internal standard solution, vortex for 10 min, add 300 µL of acetonitrile, vortex and mix for 10 min, centrifuge (13 000 r min^−1^) for 5 min, The supernatant was transferred to a 1.5 mL tube and centrifuged (13 000 r min^−1^) for 5 min at high speed. The supernatant (200 µL) was used for ultra performance liquid chromatography (UPLC)‐MS/MS analysis.

*Wound Healing Assay*: For the wound healing assay, SMMC7721 cells (1.5 × 10^5^ cells per well) were seeded in 6‐well plates and incubated in DMEM containing 10% FBS for 24 h at 37 °C, and then incubated in serum‐free DMEM for 24 h. Wounds were created by scratching the cells with a p200 pipet tip, and then the cells were washed three times with PBS. Next, cells were incubated with KGFRWR, DOX, or DOX–KGFRWR at a concentration of 2.5 µg mL^−1^ DOX/well for 24 h. The wound healing area was photographed under a microscope. The acquired images were processed using ImageJ software to quantify the wound areas. The wound areas observed at 24 h were normalized to the initial areas to count the relative migration rates for different formulations.

*In Vitro Migration and Invasion*: For migration assays, SMMC7721 cells that had been treated with KGFRWR, DOX, or DOX–KGFRWR (2.5 µg mL^−1^ DOX/well) for 24 h were trypsinized and suspended in serum‐free medium, and then 5 × 10^5^ cells were plated in the top chambers of transwells coated with Matrigel (BD Biosciences). For invasion assays, 5 × 10^5^ cells were used. Then, 500 µL of media were added to the lower chamber as a chemoattractant. After a 24 h incubation, the cells that did not migrate or invade the upper wells were removed using a cotton swab. Cells that got through the membrane and were located on the lower surface were fixed using 4% paraformaldehyde, stained with crystal violet, and quantified with a microplate reader (Leica, Germany).

*Inhibition of Liver Tumor Growth and Metastasis*: Mice bearing SMMC7721 tumors were randomly assigned to four groups (*n* = 15) and intratumor injections of saline, KGFRWR, DOX, or DOX–KGFRWR (5 mg kg^−1^ DOX) were administered three times a week for two weeks. The body weight and tumor volume were recorded and calculated three times a week for three weeks. The tumor length (*L*), which is the longest diameter of the tumor, and width (*W*), which is the diameter in the direction perpendicular to the length, were measured using Vernier calipers, and the tumor volume (*V*) was counted using the following formula: *V* = *LW* ^2^/2. Then, mice were sacrificed and photographed, and the primary tumors and the lungs were striped, and then washed with cold PBS. Primary tumors were weighed and photographed. Histological sections of the primary tumors were used for the following three purposes: first, the sections were stained with H&E to detect tumor apoptosis and necrosis; second, TUNEL staining was used to detect DNA fragmentation in the nucleus of late apoptotic cells in the tissue; and third, sections were incubated with an anticaspase‐3 rabbit antibody (1:200 dilution) followed by a horseradish peroxidase‐labeled antirabbit immunoglobulins (Abcam, UK) for color development and then counterstained with hematoxylin. For pulmonary metastatic assays, SMMC7721 cells were trypsinized, suspended in complete medium at a density of 1 × 10^7^ cells mL^−1^, and then immediately intravenously injected into the nude mice through the tail vein. Images of metastatic nodules on the lungs were obtained with a camera and nodules were counted; histological sections of lungs were stained with H&E for the histopathological examination.

## Conflict of Interest

The authors declare no conflict of interest.

## Supporting information

Supplementary
