# Supplementary material for: Drug‐Bearing Supramolecular MMP Inhibitor Nanofibers for Inhibition of Metastasis and Growth of Liver Cancer
Source: Adv Sci (Weinh). 2018 Jun 10;5(8):1700867. doi: 10.1002/advs.201700867 (PMC6097146; doi:10.1002/advs.201700867)
Supplement: Supplementary file 1 — Supplementary [file ADVS-5-1700867-s001.pdf]

Copyright WILEY-VCH Verlag GmbH & Co. KGaA, 69469 Weinheim, Germany, 2018.

Supporting Information

for *Adv. Sci.,* DOI: 10.1002/advs.201700867

Drug-Bearing Supramolecular MMP Inhibitor Nanofibers for Inhibition of Metastasis and Growth of Liver Cancer

*Yujie Ji, Yanyu Xiao, Liu Xu, Jiayu He, Chen Qian, Weidong Li, Li Wu, Rui Chen, Jingjing Wang, Rongfeng Hu, Xudong Zhang, Zhen Gu, and Zhipeng Chen**

Supporting Information

Drug-Bearing Supramolecular MMP Inhibitor Nanofibers for Inhibition of Metastasis and Growth of Liver Cancer

Yujie Ji[†]*, Yanyu Xiao*[†]*, Liu Xu*[†]*, Jiayu He, Chen Qian, Weidong Li, Li Wu, Rui Chen, Jingjing Wang, Rongfeng Hu, Xudong Zhang, Zhen Gu, Zhipeng Chen**□

Y. Ji, L. Xu, J. He, C. Qian, W. Li, L. W, R. Chen, J. Wang, Prof. Z. Chen
Department of Pharmacy, Nanjing University of Chinese Medicine, Nanjing 210023, China
Jiangsu Key Laboratory for Functional Substance of Chinese Medicine, Nanjing 210023, China
State Key Laboratory Cultivation Base for TCM Quality and Efficacy, Nanjing University of Chinese Medicine, Nanjing 210023, China
E-mail: czpcpu@sina.com
Y. Xiao
Department of Pharmacy, China Pharmaceutical University, Nanjing 210009, China

Prof. Z. Gu, X. Zhang,
Joint Department of Biomedical Engineering, University of North Carolina at Chapel Hill and North Carolina State University, Raleigh, North Carolina 27695, United States

Prof. R. Hu
Key Laboratory of Xin'an Medicine, Ministry of Education, Anhui Province Key Laboratory of R&D of Chinese Medicine, Anhui University of Traditional Chinese Medicine, Hefei, Anhui 230038, China
† These authors contributed equally to this work

1.1 Materials

Fmoc-Lys(Mtt)-OH (>98%), Fmoc-Gly-OH (>98%), Fmoc-Phe-OH (>98%), Fmoc-Arg(Pbf)-OH (>98%), Fmoc-Trp(Boc)-OH (>98%), 4-Methyl-benzhydrylamine resin (MBHA resin, 100~200 mesh, degree of substitution 0.88 mmol/g, 1% DVB), N,N,N´,N´-Tetramethyl-O-(benzotriazol-1-yl)uronium Hexafluorophosphate (HBTU, 99 %) was purchased from GL Biochem Ltd. (Shanghai, China). 1-Hydroxybenzotriazole hydrate (HOBT, 99%), N,N'-Diisopropylcarbodiimide (DIC, 99%), Ethyldiisopropylamine (DIEA, 99%), N,N-Dimethylformamide (DMF, 99%), Triisopropylsilane (TIS, 99%), (3-Hydroxy-3H-1,2,3-triazolo[4,5-b]pyridinato-O)tri-1-pyrrolidinylphosphonium hexafluorophosphate (PyAOP, 99%) was obtained from Xiya Reagent. Doxorubicin hydrochloride (DOX, 99.5%) was purchased from Shanghai Langchem Inc. Dimethyl sulfoxide (DMSO, >99.9%) was purchased from Sigma (St. Louis, MO, USA), Uranyl acetate（>99.9%) and succinic anhydride were obtained from Xi'an Dingtian Chemical Co. Ltd (Xi'an, China), Hydrochloric Acid (36–38 %, HCL) was purchased from Shanghai Ling Feng Chemical Reagent Co., Ltd (Shanghai, China), Sodium hydroxide (≥96%, NaOH) was purchased from Nanjing Chemical Reagent Co., Ltd. (Nanjing, China), Trifluoroacetic acid (≥99.5%) and HPLC grade methanol and acetonitrile were purchased from ANPEL Scientific Instrument Co. Ltd. (Shanghai, China). Formic acid was obtained from Tianjin Damao Chemical Reagent Factory (Tianjin, China). Ethyl ether (≥99.5%) was obtained from Shanghai Ling Feng Chemical Reagent Co., Ltd. (Shanghai, China). All of the chemical reagents and solvents were used as received from the commercial sources without further purification unless otherwise noted.

3-(4,5-dimethylthiazol-yl)-2,5-diphenyltetrazolium bromide (MTT), Pyrene MMP-2, MMP-9 and MMP-2/MMP-9 substrate were purchased from Sigma (St. Louis, MO, USA), phosphate-buffered saline (PBS, Gibco®) was purchased from Thermo Fisher Scientific (USA), LysoTracker® Red DND-99 and MitoTracker Red (FBS) were purchased from

Thermo Fisher Scientific (Waltham, USA). Ilomastat was purchased from Selleck Chemicals. ((Houston, USA). Trypsin, fetal calf serum and DMEM (Gibco®) was purchased from Thermo Fisher Scientific (USA). Deionized distilled water was used for the preparation of all solutions, and the other reagents were HPLC grade.

The male rats were purchased from Experimental Animal Center (Nanjing University of Chinese Medicine, China), glass capillary (inner diameter 0.9–1.1 mm, West China Medical University Instrument Factory), Pigeon centrifuge (model: 1.5 m × 12, 16000 rpm, Anting Shanghai Instrument Factory).

1.2 Preparative RP-HPLC Analysis

The peptides and the drug beacon were purified using preparative RP-HPLC with a Varian Polymeric Column (PLRP-S, 100 Å, 10 μm, 150 × 25 mm) at 25 °C on a Varian ProStar Model 325 preparative HPLC (Agilent Technologies, Santa Clara, CA) equipped with a fraction collector. A water/acetonitrile gradient 30-60 % was ran for 80 minutes containing 0.1 % v/v TFA was used as eluent at a flow rate of 20 mL/min for Dox-Fmoc-KGFRWR peptide. The absorbance peak was monitored at 480 nm.

1.3 LC-MS Characterization

The identity and purity of all synthesized materials was confirmed using LC-MS analysis. The LC-MS system composed of a LC-20AB pump, SPD-M20A diode array detector, SIL-20AC auto sampler, CTO-20A column oven (Shimadzu, Japan) and a 4000 QTRAP® mass spectrometer (AB SCIEX, USA), equipped with an Turbo ion source. Analyst software (Version 1.5.1) and Multi Quant software (Version 1.5.1) were used for data acquisition and analysis, respectively. An Agilent Technologies RP-18 (250 mm × 4.6 mm, 5 μm, Agilent Technologies, Japan) was employed for the separation of analytes at a flow rate of 1 mL/min. The column temperature was 25 °C and the wavelength was set at 480 nm. The mobile phase consisted of acetonitrile and 0.1 % trifluoroacetic acid, and a gradient method was employed

for the analysis (30-40 % acetonitrile over 0–10 min, 40–50 % methanol over 10–20 min, 50-60 % acetonitrile over 20-30 min).

1.4 Synthesis of Nir-797-KGFRWR

Briefly, 3 mg of KGFRWR was dissolved in sodium carbonate solution (pH 9.0). After complete dissolved, NIR-797-isothiocyanate was added into the solutions and the reactions were run overnight at room temperature.

1.5 *In vivo* imaging observation

The real-time tumor accumulation ability of intratumor injections of Nir-797-KGFRWR nanofiber in mice bearing SMMC7721 tumors were observed by noninvasive optical imaging systems. Nir-797 solution was as control group via intratumor injection. Then, at different time after administration, the mice were anesthetized and visualized by a Kodak multimodal imaging system (CRi Inc. Woburn, MA).

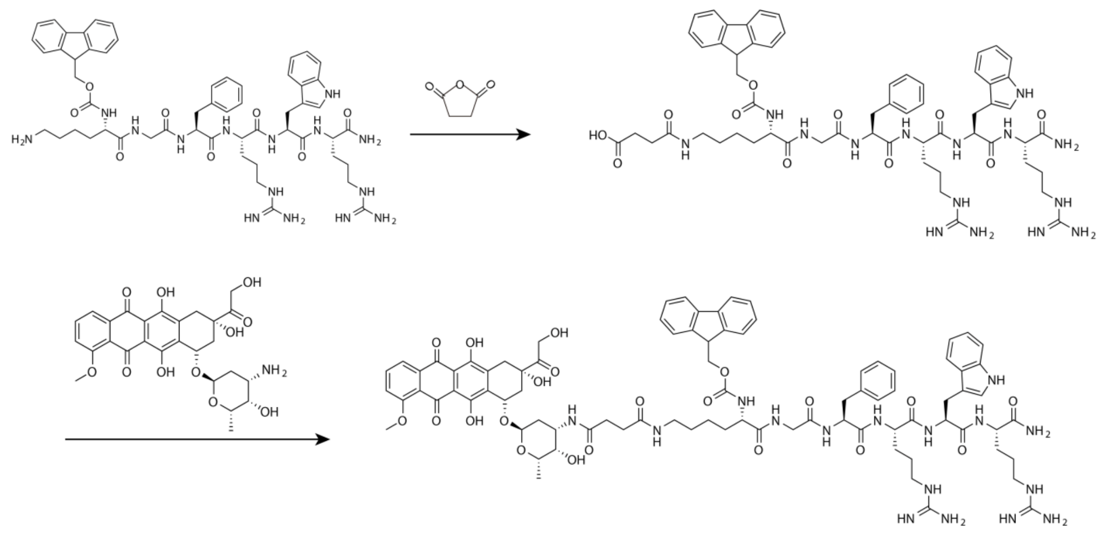

Figure S1. The synthesis of DOX-(Fmoc)KGFRWR

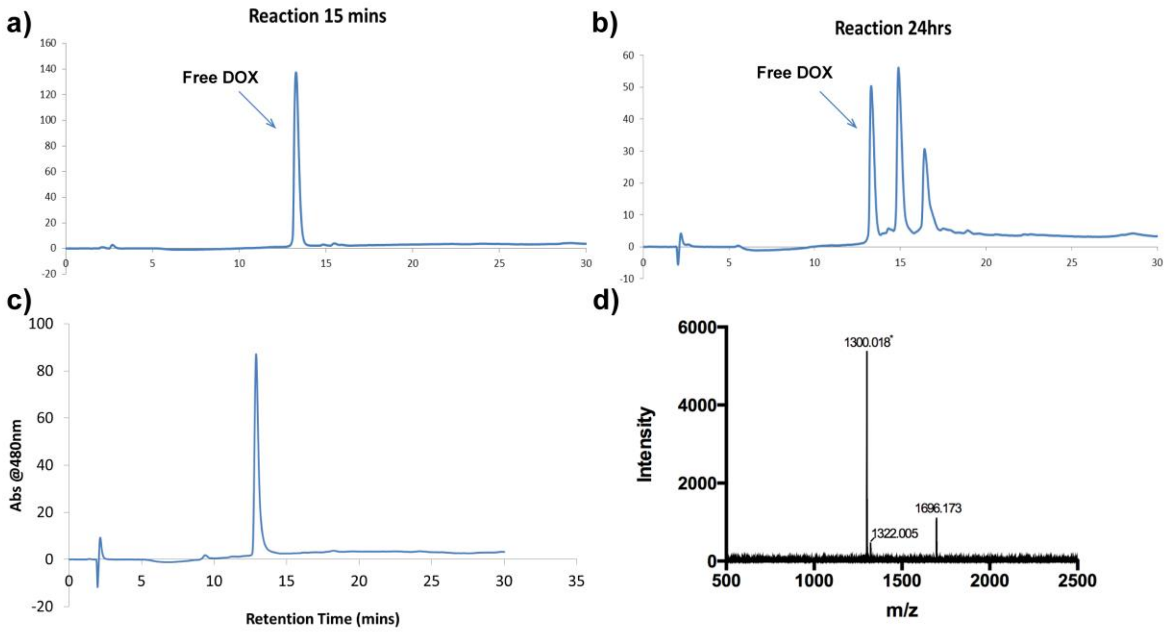

Figure S2. The high performance liquid chromatography and Mass spectrum of DOX-KGFRWR.

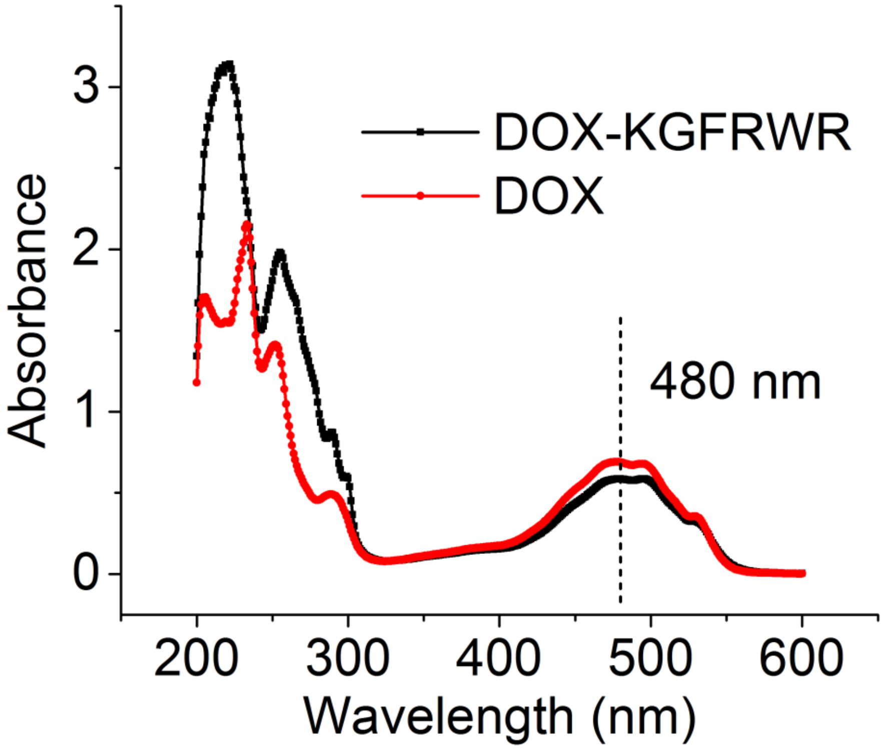

Figure S3. The UV absorption spectra of DOX-KGFRWR and DOX

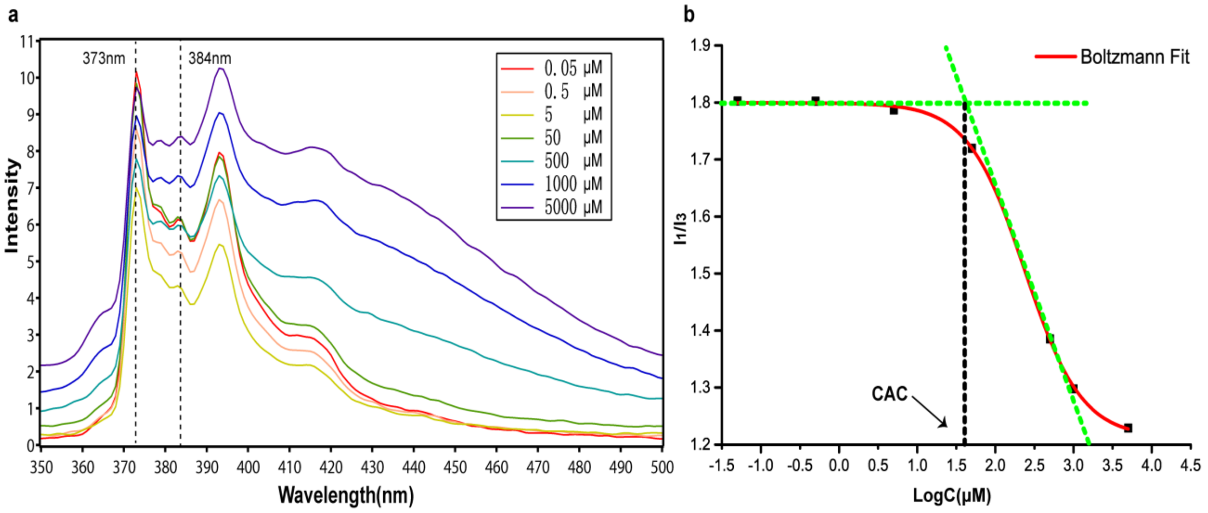

Figure S4. (a) Fluorescent emission spectra of pyrene with the increased concentration of DOX-KGFRWR and (b) the critical aggregation concentration of DOX-KGFRWR

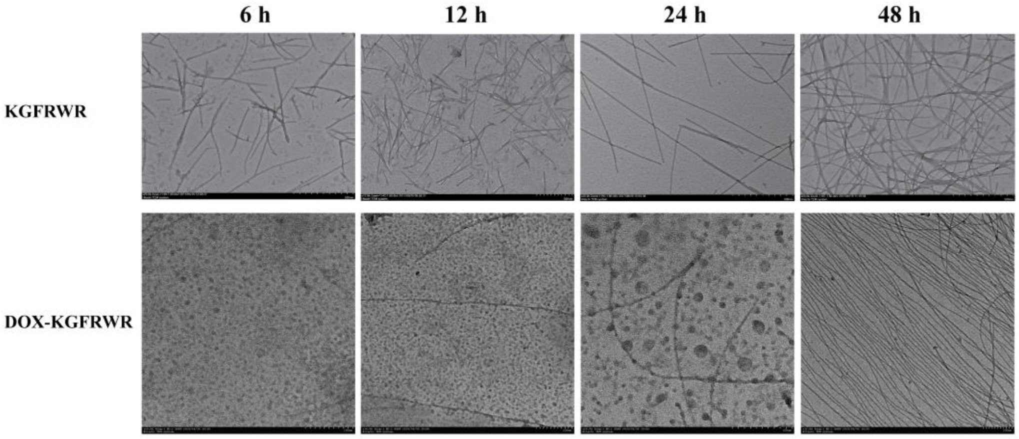

Figure S5. The TEM images of fiber forming process of KGFRWR and DOX-KGFRWR.

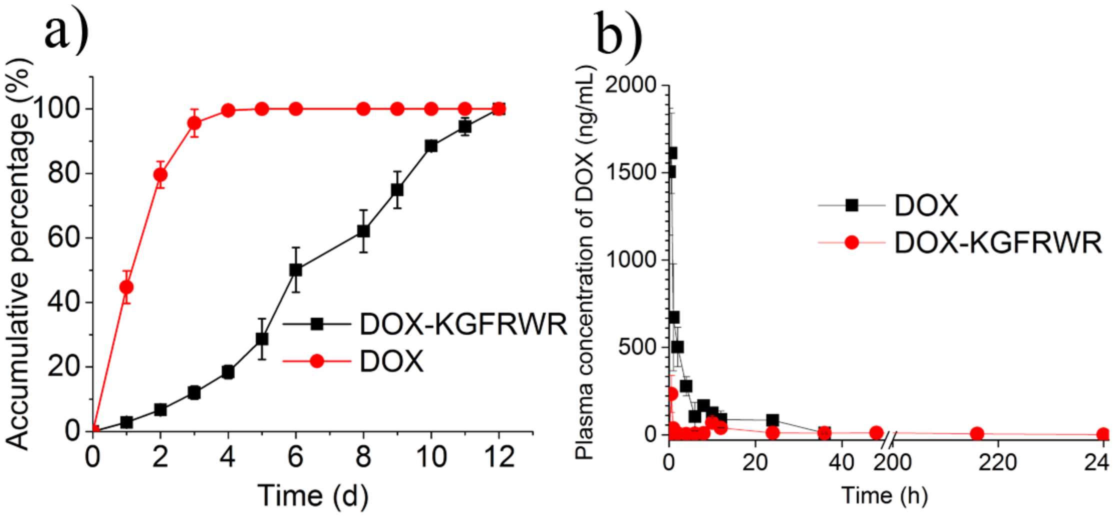

Figure S6. Release characteristics of the DOX-KGFRWR nanofibers. (a) Release profiles of DOX-based formulation from DOX solution and DOX-KGFRWR nanofibers. (b) *In vivo* pharmacokinetic study of intratumor administered DOX solution and DOX-KGFRWR nanofibers.

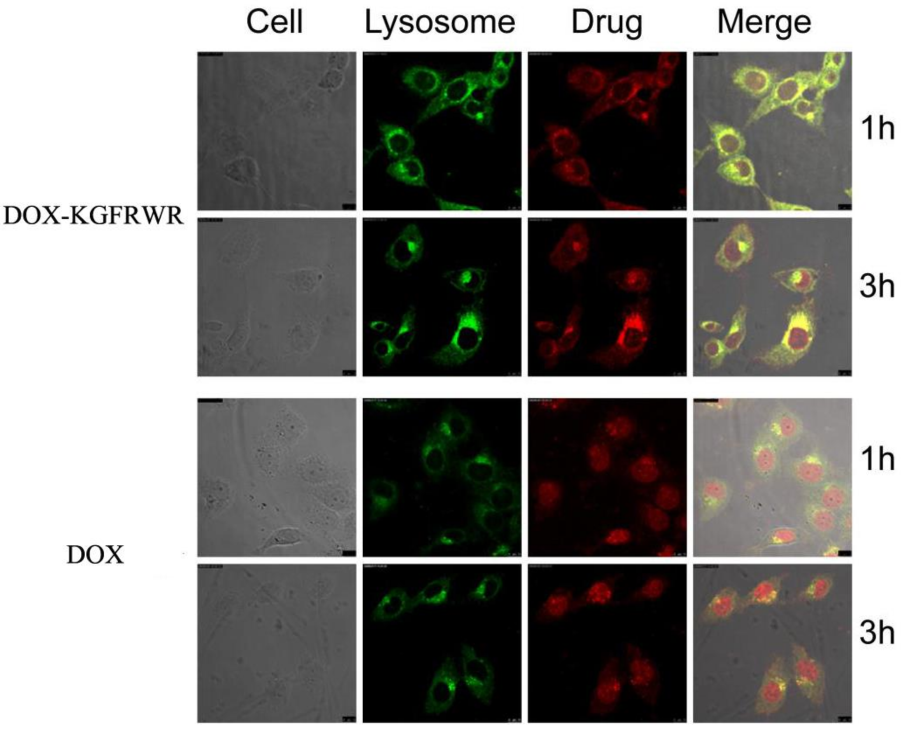

Figure S7. Lysosome localization of DOX–KGFRWR by confocal laser scaning microscopy. SMMC7721 cells were incubated with DOX–KGFRWR and DOX for 1 h and 3 h. Yellow spots in the merged pictures denote the colocalization of drug within lysosome compartments.

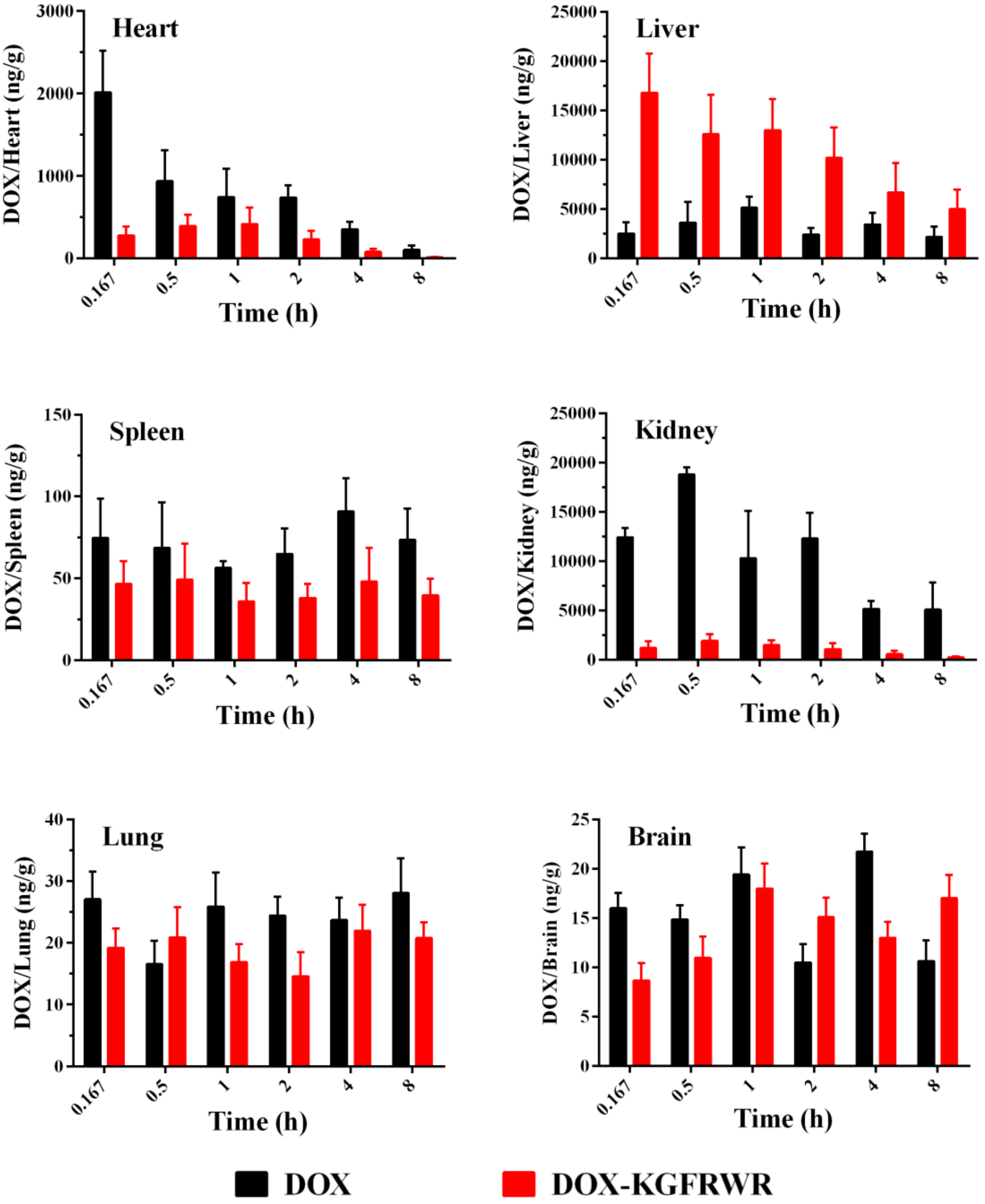

Figure S8. *In vivo* biodistribution of DOXand DOX-KGFRWR at 0.167, 0.5, 1, 2, 4, and 8 h after intratumoral administration.

Table S1. Pharmacokinetic patameters of DOX-KGFRWR gels and DOX solution in rats plasma

Parameters	DOX-KGFRWR gel	DOX solution
$AUC_{(0-t)}$(h·ng/mL)	2746.61±366.19	5356.01±769.55
$AUC_{(0-\infty)}$(h·ng/mL)	2835.49±448.24	5461.47±740.02
MRT (h)	95.28±7.65***	8.39±0.77
$T_{1/2}$ (h)	24.52±13.17*	6.90±1.20
C_{max} (ng/mL)	233.81±105.86	1639.60±249.90
T_{max} (h)	0.5±0	0.43±0.16

DOX-KGFRWR group *vs* DOX group：*P < 0.05; ***P < 0.001.
